# Enhancing Speech Emotion Recognition Using Dual Feature Extraction Encoders

**DOI:** 10.3390/s23146640

**Published:** 2023-07-24

**Authors:** Ilkhomjon Pulatov, Rashid Oteniyazov, Fazliddin Makhmudov, Young-Im Cho

**Affiliations:** 1 Department of Computer Engineering, Gachon University, Seongnam 13120, Republic of Korea; ilkhomjonpulatov@gmail.com; 2 Department of Telecommunication Engineering, Nukus Branch of Tashkent University of Information Technologies Named after Muhammad Al-Khwarizmi, Nukus 230100, Uzbekistan

**Keywords:** speech emotion recognition, CNN, LSTM, feature extraction, MFCC, spectrogram

## Abstract

Understanding and identifying emotional cues in human speech is a crucial aspect of human–computer communication. The application of computer technology in dissecting and deciphering emotions, along with the extraction of relevant emotional characteristics from speech, forms a significant part of this process. The objective of this study was to architect an innovative framework for speech emotion recognition predicated on spectrograms and semantic feature transcribers, aiming to bolster performance precision by acknowledging the conspicuous inadequacies in extant methodologies and rectifying them. To procure invaluable attributes for speech detection, this investigation leveraged two divergent strategies. Primarily, a wholly convolutional neural network model was engaged to transcribe speech spectrograms. Subsequently, a cutting-edge Mel-frequency cepstral coefficient feature abstraction approach was adopted and integrated with Speech2Vec for semantic feature encoding. These dual forms of attributes underwent individual processing before they were channeled into a long short-term memory network and a comprehensive connected layer for supplementary representation. By doing so, we aimed to bolster the sophistication and efficacy of our speech emotion detection model, thereby enhancing its potential to accurately recognize and interpret emotion from human speech. The proposed mechanism underwent a rigorous evaluation process employing two distinct databases: RAVDESS and EMO-DB. The outcome displayed a predominant performance when juxtaposed with established models, registering an impressive accuracy of 94.8% on the RAVDESS dataset and a commendable 94.0% on the EMO-DB dataset. This superior performance underscores the efficacy of our innovative system in the realm of speech emotion recognition, as it outperforms current frameworks in accuracy metrics.

## 1. Introduction

In the past decade, the rapid advancement of artificial intelligence (AI) has resulted in an increased interest in developing more advanced methods of human–machine communication. One area of research that has gained significant attention is speech emotion recognition (SER), which involves the development of algorithms and models that can detect and differentiate various emotions conveyed through speech. Human communication relies heavily on the use of emotions to communicate a speaker’s message and its purpose. Recognizing emotions in speech can help improve the accuracy of speech recognition and understanding of the spoken language, as well as enable new applications such as emotion-aware human–computer interaction.

The proficiency in precisely discerning and distinguishing emotional nuances in speech holds significant applicability across a diverse array of domains such as pedagogical environments, the entertainment sector, and healthcare. Despite the potential benefits of SER, it remains a challenging task because emotions can be subjective and difficult to detect accurately. Hence, scholars engaged in this discipline are relentlessly probing novel methodologies and strategies to enhance the precision of SER algorithms.

Conventional SER techniques typically encompass a sequence of unique phases commencing with the input and preprocessing of audio data, succeeded by the extraction of features, and culminating in emotion categorization. This process aims to distill complex emotional cues from speech, and encapsulates the core elements of SER systems. These phases are essential in building an effective SER system. In the input and preprocessing stage, audio data are first collected, and any noise or other unwanted signals are removed. The data are then converted into a suitable format for further processing. In the subsequent step, feature extraction approaches are used to produce a collection of characteristics that best reflect the emotional content of the voice signal.

Classic speech emotion recognition methods use various machine learning algorithms to classify emotions from speech signals. Support vector machines (SVMs) [1,2,3], hidden Markov models (HMMs) [4,5], and others [6,7,8] are included in these methods. Traditional SER approaches rely on handcrafted features and statistical models to extract information from speech signals and classify emotions. These methods have demonstrated limited performance and lack robustness to speech signal variations.

Deep learning-based methods have emerged as a promising approach to address these limitations and achieve state-of-the-art performance in SER tasks. Several deep learning-based SER models [9,10,11,12] have been developed over the past decade. For instance, a deep belief network-based SER model was proposed in which feature extraction and classification were performed in a single step. A hybrid deep learning-based SER model that combines convolutional neural networks (CNNs) and recurrent neural networks (RNNs) was also proposed to capture both the spectral and the temporal characteristics of speech signals.

However, several challenges, such as model accuracy, data scarcity, and generalization to unseen data, remain to be addressed in deep learning-based SER models. Numerous studies have attempted to develop optimized SER models by utilizing one or two sources of features. However, there is currently no established set of features that has been experimentally proven to be appropriate for building an effective SER model. Despite numerous exploratory endeavors, a uniform agreement pertaining to the selection of features that would yield optimal performance in SER models is yet to be established.

The decision on the most appropriate features for SER is still a subject of ongoing debate and investigation in this domain, underscoring the complexity and multifaceted nature of speech emotion recognition. Therefore, further research is required to determine the ideal combination of features for building an efficient SER model. Mel-frequency cepstral coefficient (MFCC)-based models have been used in various speech recognition applications, including keyword spotting, speaker identification, and speech-to-text transcription. One of the strengths of MFCC-based models is their computational efficiency and suitability in real-time applications. However, these models may not capture the full semantic meaning of speech because they only capture acoustic characteristics.

Thus, in this study, we attempted to use the advantages of Speech2Vec, which can capture both the acoustic and the semantic characteristics of speech, as well as MFCCs. In addition, frequency and time information was obtained via spectrograms to detect the emotional characteristics of speech and changes in emotion over time. Overall, we endeavored to construct a novel model for SER based on spectrograms and semantic feature encoders that enhance performance accuracy by considering the notable deficiencies in existing approaches and remedying them.

To obtain useful features for speech recognition, this study utilized two distinct methods. First, a fully CNN model was utilized to encode speech spectrograms. Second, a novel MFCC feature extraction technique was utilized and combined with Speech2Vec to encode semantic features. These two types of features were processed separately and subsequently fed into a long short-term memory (LSTM) and a fully connected layer for additional representation. This study presents several significant contributions that should be highlighted.

- First, it introduced a new methodology for SER that achieved superior accuracy compared to existing baseline models. This novel approach provides a promising direction for future SER research.
- Second, innovative techniques were utilized to extract semantic features from audio signals. This feature extraction process involved combining MFCCs with Speech2Vec to create a more meaningful representation of speech data. These semantic features contribute significantly to the accuracy of the SER model.
- Third, the study improved the model complexity by using a deep learning architecture that included an LSTM and a fully connected layer. This method enabled the production of an embedding of a predetermined length for an input piece, which is necessary for activities involving speech recognition. This fixed-length embedding simplifies the processing of speech signals and improves the accuracy of SER.
- Overall, the contributions of this study have advanced our understanding of SER and provided valuable insights into developing more effective speech recognition models. These findings have important implications for a range of applications, including emotion recognition in human–robot interaction, speech therapy, and mental health diagnosis.

The ensuing segments of this manuscript unfold in the following manner: Section 2 furnishes an exhaustive review of contemporary research on SER modeling, focusing on the employment of MFCCs, semantic features, and other profound learning models. Section 3 and Section 4 proffer an in-depth delineation of the proposed SER model, along with the empirical findings that corroborate its effectiveness, and juxtapositions with established benchmarks. These sections aim to provide readers with a thorough understanding of the model’s design and capabilities, as well as its relative performance in the field of SER. Section 5 provides a concluding summary and discusses potential future research directions. The article concludes with a reference list that includes several contemporary publications on SER.

## 2. Literature Review

Understanding emotions in speech is a complex task that requires significant effort from researchers to develop highly effective models using algorithms. This is because emotions may be communicated via speech in various ways, including changes in tone, pitch, loudness, rhythm, and other speech-related characteristics. Currently, there are multiple studies [13,14,15,16] that focused on identifying and analyzing speech features to detect the emotions of individuals. These studies aimed to effectively classify the detected features and accurately determine the emotional state of the speaker. The accurate and effective extraction of relevant characteristics, as well as the high correlation among these features, are critical elements that significantly affect the effectiveness of the emotion detection system. Contemporary SER approaches have been positively affected by the introduction of several innovative feature extraction methods [17,18,19,20]. In one study [17], a deep neural network model for SER that could simultaneously learn both MelSpec and GeMAPS audio features was proposed. The three components of the model are the learning of MelSpec in picture format, learning of GeMAPS in vector format, and combining the two to predict emotions. Moreover, the study conducted by Lalitha et al. [21] explored the effectiveness of different feature extraction modules according to cepstral coefficients for detecting emotions in speech. Cepstral coefficients, which reflect the spectral envelope of a speech signal, are common characteristics of speech processing. This study investigated the use of different cepstral coefficient-based features, such as MFCCs, linear predictive cepstral coefficients (LPCCs), and perceptual linear predictive cepstral coefficients (PLPCCs), for emotion recognition in speech. The authors of [22] suggested a new approach to address the issue of long-term dependence vanishing in RNNs. Specifically, they introduced a novel method using linear predictive Meir frequency cepstrum coefficients and bidirectional LSTM to recognize dance emotions. Several studies [23,24,25] have demonstrated that combining MFCCs with other feature sets can enhance emotion recognition accuracy.

On the other hand, semantic feature encoders are deep learning models that encode speech signals into high-dimensional semantic vectors that capture the meaning of the speech. There are several recent semantic feature encoder-based SER models [26,27,28,29]. Kakuba et al. [29] formulated a deep learning-based methodology that can concurrently acquire spatial, temporal, and semantic representations in a unified manner within a local feature learning block. This technique merges the aforementioned representations into a latent vector, which subsequently serves as the input for the global feature learning block. Moreover, Yoon et al. [30] suggested a deep dual recurrent encoder that incorporates both text and audio data by employing two separate RNNs to encode the text and audio sequences and subsequently merging the information from both sources to enhance the performance of emotion classification in emotional dialogues. The method [31] first feeds the aligned multimodal features into a sequential model to enhance the accuracy of multimodal feature representations for emotion identification by learning the alignment between voice frames and text words.

Overall, combining multiple features can enhance the robustness and generalization of SER models because it reduces the impact of individual feature biases and improves the ability of the model to handle different types of emotions and speech contexts.

## 3. Proposed System

**Figure 1:**
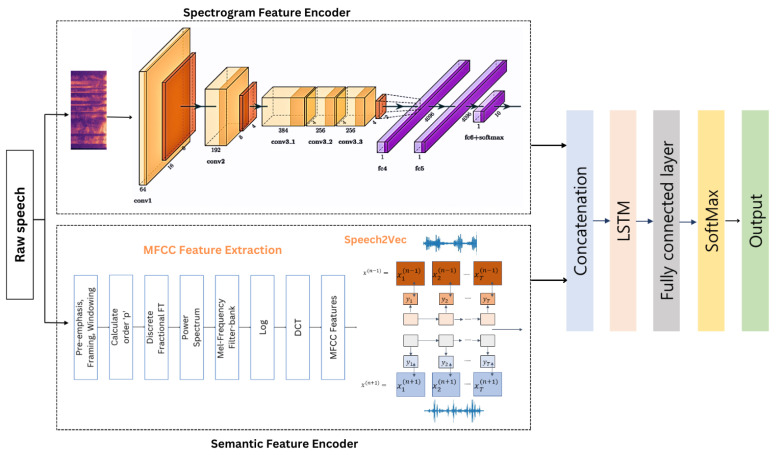
Modeling process of the proposed system.

This section elucidates the intricacies of the proposed framework, specifically architected for the recognition of emotions in speech. The schema encompasses two principal constituents, each indispensable in delivering an accurate prognosis of the speaker’s emotional disposition. The comprehensive process of modeling is depicted in Figure 1, which demonstrates the chronology of steps involved in the model’s execution. The disparate components of the model operate synergistically to accomplish the objective of speech emotion recognition. The model is adept at evaluating and interpreting a plethora of acoustic facets intrinsic to speech signals, inclusive of aspects such as duration, intensity, and pitch, to deduce the latent emotional state of the speaker. On the whole, the proposed model embodies a holistic and robust methodology to speech emotion recognition, possessing the capacity for application in a vast spectrum of real-world situations. This degree of versatility enhances the model’s utility and positions it as a powerful tool in the evolving field of speech emotion recognition.

### 3.1. Semantic Feature Encoder

#### 3.1.1. MFCC Feature Extraction

**Figure 2:**
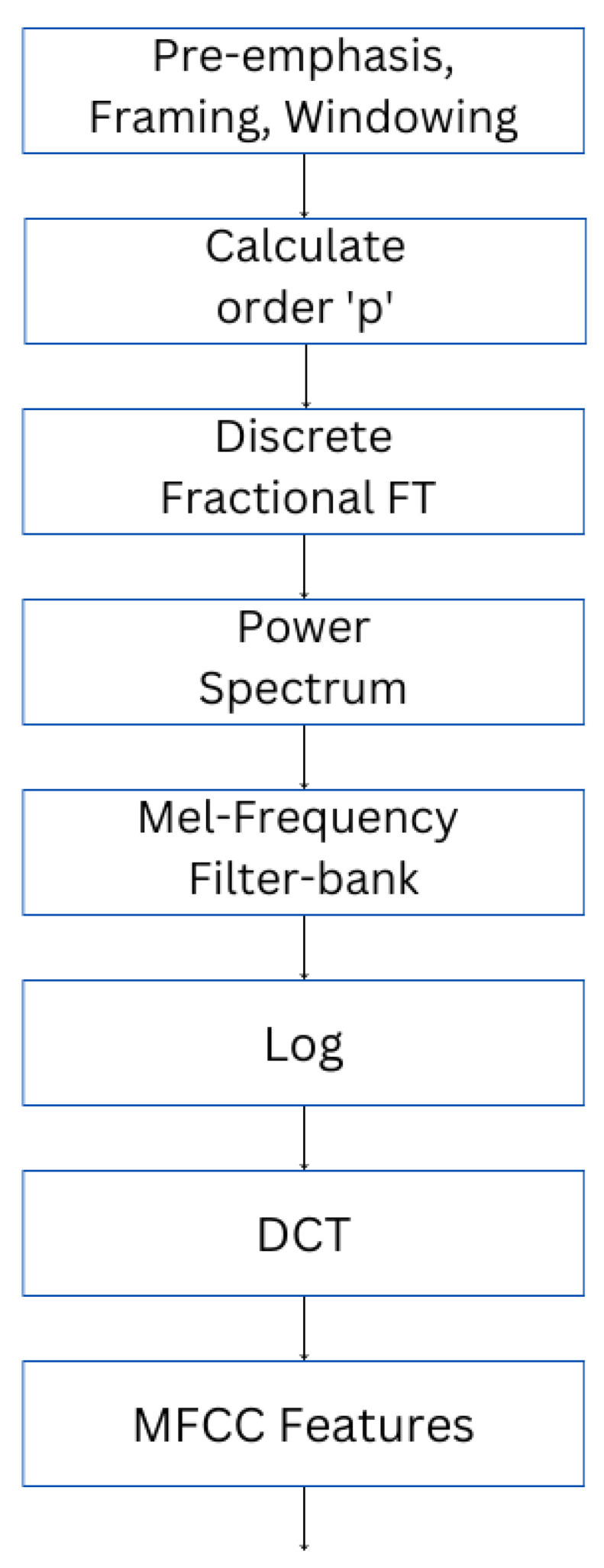
MFCC feature extraction procedure.

The utilization of mathematical computations to depict the auditory mechanism of the human ear is a characteristic of the MFCC method, which can attain remarkable recognition rates. Consequently, this study adopted the MFCC (Figure 2) as a prominent feature for the recognition of speech emotions [32]. Despite the utility of the traditional MFCC parameter in cepstral analysis, it only encompasses the invariant features of speech parameters. Hence, this study aims to augment the analysis by computing a differential spectrum that incorporates dynamic features. First, the AF approach is utilized to ascertain the optimal order p of the fractional Fourier transform (FrFT), which is subsequently operationalized in the extraction of MFCC features.

The overarching procedure can be delineated as follows:

1. Regarding the preprocessing of speech signals, the initial step involved pre-emphasizing the primary speech signal via filtration using $H\left(z\right)=\left(1-0.97z^{-1}\right)$. The signal was then divided into frames of 20 ms duration and 10 ms phase shift. The application of a 20 ms frame length and a 10 ms shift adeptly caters to the complex demands of speech signal processing. This configuration not only ensures ample data capture for reliable spectral analysis but also enables frequent updates to track the rapid changes inherent in speech signals. Finally, Hamming windows were applied to the frames to improve the analysis.
2. To ascertain the ideal order “p” for each frame of the preprocessed speech signal, the ambiguity function (AF) was employed and obtained using Equation (1).
  (1) $$A_{z}\left(\tau ,v\right)=\int_{-\infty }^{\infty }z(t+\frac{\tau }{2})z^{*}(t-\frac{\tau }{2})\exp(-j2\pi \mathrm{vt})\mathrm{dt}.$$
3. Applying the optimal order “p” (p = 1.03), the discrete fractional Fourier transform (DFrFT) was executed on each frame of the speech signal, followed by squaring the resultant output to obtain the energy spectrum.
4. The energy spectrum was processed using a Mel filter bank that operates in a uniformly spaced frequency range, thereby transforming the linear frequency scale into a Mel frequency scale. Subsequently, logarithmic compression was applied to the output. The Mel scale and frequency have a specific interrelation, which can be denoted as follows:
  (2) $$\mathrm{Mel}\left(f\right)=2595\mathrm{lg}\left(1+\frac{f}{700}\right).$$
5. To derive a set of 39 acoustic features, the logarithmic energy of the filter bank was first subjected to discrete cosine transform (DCT) to yield 13 static MFCCs. Additionally, the first- and second-order differentials of the MFCCs were computed to obtain the first- and second-order dynamic features. The equation used to compute the dynamic features is as follows:
  (3) $$d_{t}=\frac{\sum_{k=1}^{K}k\left(C_{t+k}-C_{t-k}\right)}{2\sum_{k=1}^{K}k^{2}}.$$

Using K = 2, the first-order dynamic features ($d_{t}$) can be computed on the basis of the cepstrum coefficient ($C_{t}$). Similarly, by substituting $C_{t}$ with $d_{t}$, the equation can be used to calculate the second-order dynamic feature.

#### 3.1.2. Speech2Vec

The structure of the neural network, specifically named Speech2Vec, was deliberately employed to master predefined-length vector representations of audio segments gleaned from a speech corpus. These vectors are strategically positioned in proximity to other vectors within the embedding space, provided the spoken words they correspond to bear semantic similarity. This arrangement fosters an intricate relationship among vectors, effectively mapping semantically related words closer together, thereby creating an organized and intuitive representation of speech. The semantic information that these vectors carry is connected to the spoken words in the audio. The process of learning these vector representations involves training the Speech2Vec model using a large corpus of speech data. During training, the model learns to map each audio segment to a high-dimensional vector representation that captures the semantic features of the words uttered in the segment. The model achieves this by using a series of layers that extracts relevant features from the audio signal and subsequently transforms them into a predefined-length vector characterization. The underlying architecture of Speech2Vec is built on the RNN encoder-decoder model. The model incorporates the skip-gram methodology, which is a popular approach for training word embeddings, and which is specifically designed to learn the vector representations of words directly from speech data. By learning embeddings from speech, Speech2Vec can leverage the additional semantic information contained within the audio signal that is not present in text data.

The aim Is to secure a uniform embedding of an audio fragment corresponding to a specific word, represented by a sequence of acoustic characteristics, such as MFCC and $x=\left(x_{1},\;x_{2},\;x_{3}\ldots ,\;x_{T}\right)$, where $x_{t}$ denotes the acoustic feature at time t, and T is the length of the sequence. The objective is to produce a word embedding that reflects the semantic meaning of the initial audio segment to a certain extent.

The fundamental architecture of Speech2Vec is based on an RNN encoder–decoder framework consisting of two components: an encoder RNN and a decoder RNN, as described in previous studies [18,19]. The encoder reads each symbol $x_{t}$ of an input sequence $x=\left(x_{1},\;x_{2},\;x_{3}\ldots ,\;x_{T}\right)$ in sequence and updates the hidden state $h_{t}$ of the RNN accordingly. Once the final symbol $h_{T}$ is processed, the corresponding hidden state $h_{T}$ is treated as the learned representation of the entire input sequence. The decoder then generates an output sequence $y=\left(y_{1},\;y_{2},\;y_{3}\ldots ,\;y_{T^{\prime }}\right)$ sequentially, with T and T’ potentially differing, by initializing its hidden state using $h_{T}$.

**Figure 3:**
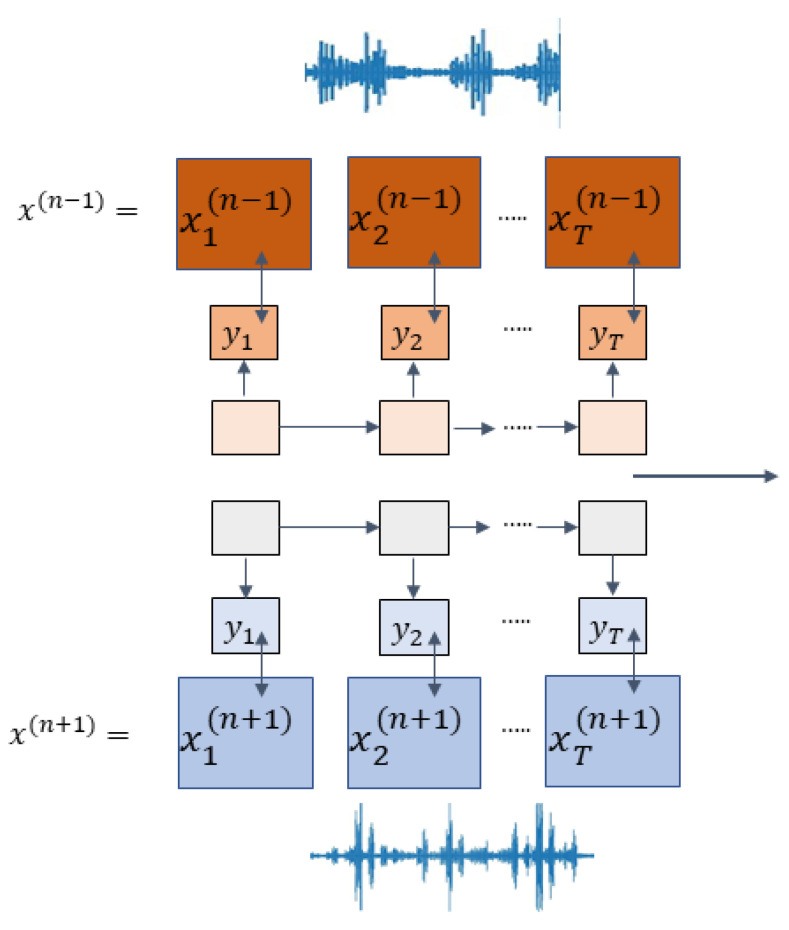
Speech2Vec via skip-gram.

The concept underlying the training methodology of Speech2Vec Is based on the use of skip-grams (Figure 3). For each audio segment $x^{\left(n\right)}$ in a given speech corpus, which corresponds to a word, the model is trained to predict audio segments $\left\{x^{\left(n-s\right)},\;\ldots ,\;x^{\left(n-1\right)},\;x^{\left(n+1\right)},\;\ldots ,\;x^{\left(n+s\right)}\;\right\}$ that correspond to nearby words within a specified range “s”. During the training process, the encoder receives $x^{\left(n\right)}$ as the input and produces a fixed-dimensional vector representation $v^{\left(n\right)}$ by encoding it. The decoder then maps $v^{\left(n\right)}$ to various output sequences $y^{\left(i\right)}$, where i ∈ {n − s, …, n − 1, n + 1, …, n + s}. The model is trained by minimizing the general mean squared error between the output sequences and their corresponding nearby audio segments, which is calculated as $\underset{i}{\sum }\|x^{\left(i\right)}-y^{\left(i\right)}{\|}^{2}$. The idea behind this approach is that the encoded vector representation $v^{\left(n\right)}$ should contain sufficient semantic information about the current audio segment $x^{\left(n\right)}$ to successfully decode nearby audio segments. Once the training process is complete, $v^{\left(n\right)}$ is used as the word embedding for $x^{\left(n\right)}$.

### 3.2. Spectrogram Feature Encoder

**Figure 4:**
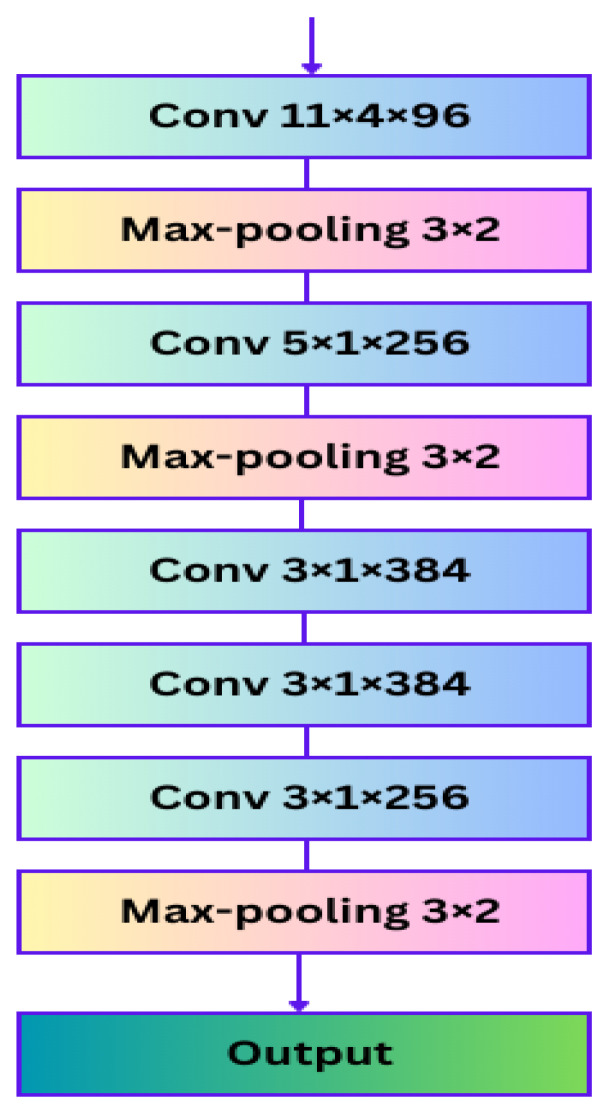
Spectrogram feature encoder.

A fully convolutional neural network (FCNN) was used in this component of the system to fulfill the goal of preventing the loss of essential features, which is the aim of this particular segment. The FCNN eliminates the need for a segmentation process to handle speech data of various lengths. In addition, various models [33,34,35] based on deep learning have been constructed to generate efficient utterance characteristics and attain higher levels of accuracy. In one study [34], raw speech spectrograms were partitioned into specific-sized chunks to conform to CNN specifications. Consequently, the feeling description of the full relevant speech was distributed throughout all chunks of the segmented utterance. Therefore, it is not logical to suppose that the segmented speech pieces do not reflect the overall emotion being communicated. We believe that breaking the speech spectrogram into pieces results in a change in the coherence of the speaker’s speech, which in turn signals a shift in emotion. Thus, the proposed model integrates FCNN as a component to minimize information loss and handle varying lengths of speech spectrogram. Moreover, a data input of any size can be processed by FCNN, which subsequently generates an output that is appropriately understood and learned. The spectrogram feature encoder of the proposed model is a variant of FCNN (shown in Figure 4) taken from AlexNet [35] but without any completely linked layers.

The FCNN consists of five convolutional layers and utilizes local response normalization after the first and second layers, as well as the ReLU activation function after each convolutional layer. ReLU avoids saturation because it does not require input data normalization. Learning occurs within a neuron if ReLU receives positive feedback from specific training datasets. However, we strive for generalization using the local normalization approach expressed in Equation (4), with hyperparameters of local response normalization N; all kernels of the corresponding layer use constant values of β = 0.75, m = 5, l = 2, and α = 0.0001.

(4) $$b_{g,h}^{u}=\frac{a_{g,h}^{u}}{{\left(l+\alpha \sum_{v=\max\left(0,\;u-\frac{m}{2}\right)}^{\min\left(N-1,\;u+\frac{m}{2}\right)}{\left(a_{g,h}^{v}\right)}^{2}\right)}^{\beta }}.$$

The convolutional layer settings are expressed as “kernel size” × “stride size” × “channels”. After local response normalization and pooling, the second and third convolutional layers receive input with parameters of 5 × 1 × 256 and 3 × 1 × 384, respectively. The first convolutional layer, on the other hand, has settings of 11 × 4 × 96. The fourth layer has the same parameters as the third layer, whereas the fifth layer has parameters of 3 × 1 × 256. Pooling layers in CNNs aggregate the results of adjacent neuronal groups in the same kernel map. The FCNN encoder produces a three-dimensional array of sizes O × P × Q, where each dimension represents a different aspect of the data. In the context of the spectrogram, the “O” and “P” dimensions represent the frequency and time domains, respectively, while the “Q” dimension represents the size of the channel. The output is assumed to be a set with “l” components, where l = P × O, with P and O denoting the lengths of the dimensions representing the frequency and time domains, respectively. Equation (5) expresses each component “L” as a Q-dimensional vector that encodes a specific segment of the input speech spectrogram.

(5) $$S=\left\{s_{1},\cdots s_{l}\right\},\;s_{u}\in {\mathbb{R}}^{Q}.$$

Ultimately, the output of the speech feature encoding, obtained from the spectrogram feature encoder module of the suggested model, is combined with the output derived from the semantic feature encoder module.

## 4. Experiment

### 4.1. Datasets

Developing SER models requires large amounts of labeled training data, which can be time-consuming and expensive to obtain. Therefore, the effectiveness of the proposed model was demonstrated by evaluating it on the RAVDESS [36] and EMO-DB [37] datasets, which are publicly available and widely used in the research community for studying speech emotion recognition. These datasets were used to compare the proposed model with existing models and demonstrate its superior performance.

#### 4.1.1. RAVDESS

**Figure 5:**
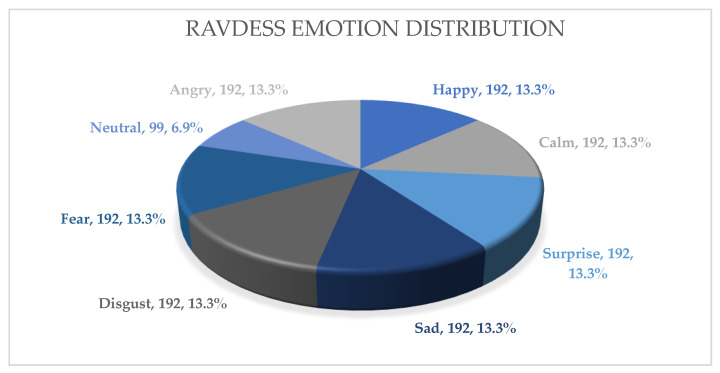
RAVDESS dataset emotion distribution.

The Ryerson Audiovisual Database of Emotional Speech and Song (RAVDESS) is a dataset that contains emotional speech and song recordings publicly available for research purposes. The RAVDESS dataset includes a variety of emotional expressions, such as neutral, calm, happy, sad, angry, fearful, surprised, and disgusted, which are spoken in both English and French. The speech recordings are monologues, and each actor speaks the same set of 13 sentences, including statements and questions with different emotional expressions. The dataset is highly reliable and valid, with good inter-rater agreement and high accuracy in predicting emotional expressions. One of the advantages of this dataset is its diversity of emotions and actors, which enables the development of more robust and generalizable emotion recognition models. Additionally, the dataset includes both speech and song recordings, which enables the study of emotion recognition in different types of audio signals. The data gathering involves a group of 24 skilled individuals, comprising an equal number of males and females, ensuring a balanced representation of genders. The speech dataset includes a total of 1440 files, obtained by multiplying 60 trials per actor with 24 actors. These audio files are encoded in the WAV raw audio file format, with a 16 bit bitrate and a sampling rate of 48 kHz. Each emotion in the RAVDESS dataset (Figure 5) is represented by an equal distribution of 192 samples, except for the “neutral” emotion, which has a count of 96 samples.

Overall, the RAVDESS dataset is a valuable resource for researchers in the field of emotion recognition and has contributed to the development of automatic emotion recognition systems using machine learning algorithms. The emotions are relatively evenly distributed in the speech subset of the RAVDESS dataset.

#### 4.1.2. EMO-DB

**Figure 6:**
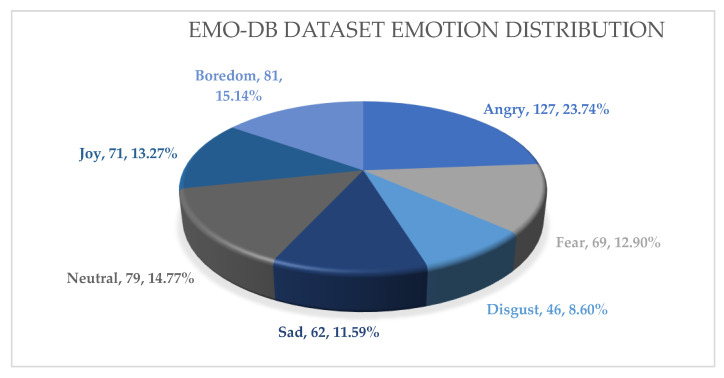
EMO-DB dataset emotion distribution.

The Berlin Emotional Speech Database (EMO-DB) is a dataset that contains emotional speech recordings widely used in research on automatic emotion recognition. The EMO-DB, a database of emotions from Berlin, consists of 535 utterances (Figure 6) captured by 10 actors, including five males and five females. Each actor performed readings of predetermined sentences while expressing various emotions such as anger, fear, boredom, disgust, happiness, neutrality, and sadness. The sentences were selected to be semantically neutral, and the emotional expressions were elicited using different methods, including role-playing, imagery, and recall of emotional experiences. The utterances in the EMO-DB typically last for approximately 2 to 3 s and have a sampling rate of 16 kHz. The dataset also includes detailed annotations of the recordings, including the onset and offset times of each emotion, as well as information about the speaker and the recording conditions. One of the advantages of the EMO-DB dataset is its controlled recording conditions, which ensure high-quality recordings and minimize the variability in the acoustic characteristics of the speech signal. Additionally, the dataset contains a wide range of emotions and involves different speakers, which enables the development of more robust and generalizable emotion recognition models.

In order to secure an unbiased assessment of our model employing the RAVDESS and EMO-DB datasets, we embraced a comprehensive end-to-end training strategy by restructuring the original data, as expounded in [38]. Specifically, we partitioned the data into training and test subsets, apportioning 80% and 20% of the samples, respectively, to the training and testing divisions. This approach ensures that the model is trained on a substantial portion of the data while also reserving a significant subset for evaluation, thereby facilitating a rigorous and comprehensive assessment of its performance. This produced 1152 training samples and 288 testing samples for the RAVDESS dataset. Similarly, for the EMO-DB dataset, we allotted 428 training samples and 107 testing samples.

Contrary to the methodology delineated in [39], we did not incorporate the 10-fold cross-validation technique in our investigation. This decision was made due to the practical difficulties involved in implementing cross-validation on deep learning models, which would have required a substantial amount of time and computational resources.

### 4.2. Evaluation Metrics

In the field of speech emotion recognition, models are typically evaluated using three common metrics: precision, recall, and accuracy. These metrics are widely used and accepted by the SER community.

#### 4.2.1. Precision

Precision is a key evaluation metric used to assess the accuracy of correctly detected actual utterances in a given model. It measures the proportion of correctly predicted positive instances (i.e., true positives) out of all instances predicted as positive, taking into account both true positives (TP) and false positives (FP). The precision metric is calculated using Equation (6).

(6) $$\mathrm{Precision}=\frac{\mathrm{TP}}{\left(\mathrm{TP}+\mathrm{FP}\right)},$$

where TP represents the true positives, which are the instances correctly identified as positive by the model, and FP represents the false positives, which are the instances incorrectly classified as positive by the model. Precision provides insights into the model’s ability to avoid false positives, indicating how reliable and accurate the positive predictions are. A higher precision score indicates a lower rate of false positives, suggesting that the model is more precise and selective in identifying actual positive instances.

#### 4.2.2. Recall

Recall is an important evaluation metric that measures the ability of a proposed model to accurately detect positive instances. It provides insight into the number of positive instances that are correctly identified by the model. Recall is calculated using Equation (7).

(7) $$\mathrm{Recall}=\frac{\mathrm{TP}}{\left(\mathrm{TP}+\mathrm{FN}\right)},$$

where TP represents the true positives, which are the positive instances correctly identified by the model, and FN represents the false negatives, which are the positive instances incorrectly classified as negative by the model. By calculating recall, we can assess the model’s ability to capture all positive instances in the dataset. A higher recall score indicates a greater ability of the model to accurately detect positive instances, suggesting a more comprehensive coverage of the positive class by the model’s predictions.

#### 4.2.3. Accuracy

The accuracy with which a sound class may be determined from a whole speech signal is a crucial assessment measure. It reveals how often and how quickly certain categories of speech sounds are recognized and labeled across the whole signal. This includes phonemes, words, and even emotions. A higher accuracy score suggests that the speech recognition or classification system is able to capture and discriminate among different classes of sounds with greater precision and reliability. For applications such as voice recognition, speaker identification, and emotion detection, knowing how well a model or algorithm predicts the proper sound classes in a spoken signal is crucial. Accuracy is calculated using Equation (8).

(8) $$\mathrm{Accuracy}=\frac{\mathrm{TP}+\mathrm{TN}}{\left(\mathrm{TP}+\mathrm{TN}+\mathrm{FP}+\mathrm{FN}\right)}.$$

### 4.3. Implementation Environment

The methodology advocated in this investigation was materialized using distinctive hardware and software configurations, the particulars of which are delineated in Table 1. This tabulation offers an exhaustive synopsis of the components that were marshalled to execute the proposed strategy. This clear inventory of resources used provides transparency into the computational backbone of our methodology, ensuring replicability and further research. Using the specified hardware and software, we successfully conducted the study and obtained the desired outcomes.

Our system was trained and tested for 100 epochs and 32 batch sizes using RTX 3090 Ti 24 GB and AMD EPYC 7543 32-Core Processor with Linux Ubuntu and 128 GB RAM. The Adam optimizer, with a learning rate of 0.001 and a learning rate decay with a factor of 10 every 20 epochs, was used for hyperparameter tuning.

### 4.4. Results

In order to articulate the extent of the proposed methodology’s superiority over the competitor models, we juxtaposed it against some benchmarks. The outcomes of our prognostications are depicted in Table 2. The chosen models, along with the one propounded in this study, are all competent schemas for SER assignments. Nevertheless, our system excelled beyond the selected models in executing SER tasks, particularly when amalgamated with semantic data. This achievement underscores the advanced capabilities of our model, demonstrating its potential to outstrip existing methods in the realm of speech emotion recognition.

1. The authors of [40] proposed a unique SER model in response to the shortcomings of prior SER approaches, such as accuracy deficiencies in intricate situations and ineffective learning of features from compound acoustic signals. This model adopts a data augmentation strategy before feature extraction, and the resulting 273 features are then supplied to a transformer model, thereby significantly improving emotion detection capabilities.
2. The authors of [41] proposed a methodology for SER that leverages MFCC and a one-dimensional convolutional neural network with the aim of diminishing computational complexity. The approach involves the use of various acoustic properties to present collaborative low-order and high-order features and the development of a lightweight one-dimensional deep convolutional neural network to streamline the deep learning frameworks for SER.
3. In [42], a new hybrid architecture was introduced to enhance the accuracy of speech emotion recognition. The proposed method involves extracting acoustic features such as RMS, MFCC, and zero-crossing rate, as well as obtaining deep features from spectrogram images using a pretrained ResNet101 network. These features are combined to create a hybrid feature vector, which is then refined using the ReliefF algorithm for efficient feature selection. Finally, the support vector machine is employed for accurate classification.
4. The authors of [43] suggested a proposal that involves utilizing a bagged ensemble consisting of support vector machines with a Gaussian kernel, which incorporates a combination of spectral features that are processed, reduced, and proven to deliver superior performance compared to individual estimators, thereby offering a suitable solution for the given problem.

Table 2 compares the accuracy percentages of different SER models when applied to the RAVDESS and EMO-DB datasets. The proposed model showcased superior performance compared to the others, achieving the highest accuracy on both datasets: 94.8% on the RAVDESS dataset and 94.0% on the EMO-DB dataset. On the other hand, the M-DCNN [42] model demonstrated relatively high performance with 94.18% accuracy on the RAVDESS dataset and 93.31% accuracy on the EMO-DB dataset. The primary advantage of our proposed system lies in its ability to comprehend the full semantic meaning of speech. Unlike the M-DCNN, which solely focuses on acoustic characteristics, our approach leverages the power of Speech2Vec to capture both acoustic and semantic characteristics of speech, resulting in a more comprehensive understanding of speech signals. The models by Bilal et al. [42] and Bhavan et al. [43] had a lower performance on the RAVDESS dataset with 79.41% and 75.69% accuracy, respectively, but they managed to reach 90.21% and 92.45% accuracy on the EMO-DB dataset.

**Table 2:** Comparison of model performances.

Models	Accuracy (%)	
	RAVDESS	EMO-DB
Tran [40]	-	93.0
M-DCNN [41]	94.18	93.31
Bilal et al. [42]	79.41	90.21
Bhavan et al. [43]	75.69	92.45
The proposed	94.8	94.0

**Figure 7:**
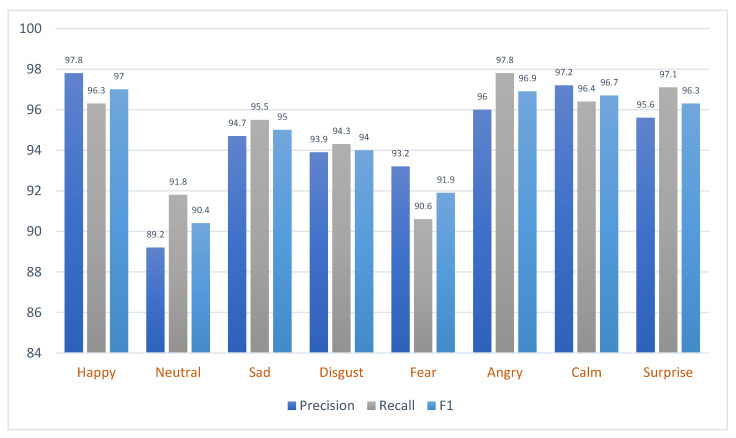
The performance of the proposed model on the RAVDESS dataset.

**Figure 8:**
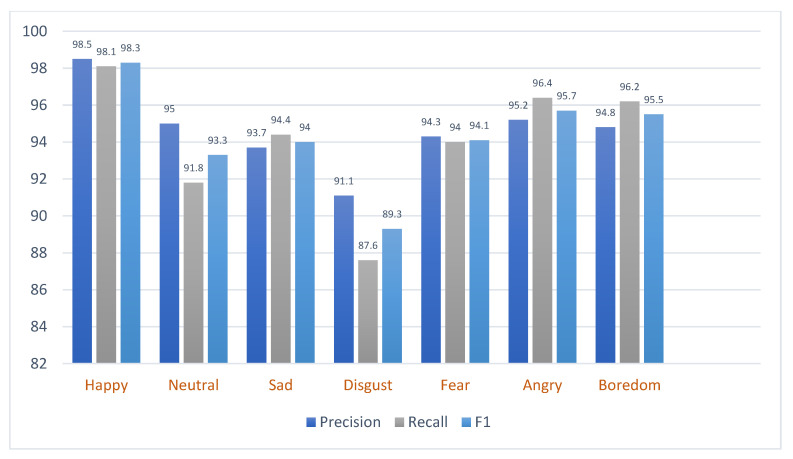
The performance of the proposed model on the EMO-DB dataset.

Moreover, Figure 7 and Figure 8 provide a detailed breakdown of the precision, recall, and F1-score for different emotions as identified by the model when applied to the RAVDESS and EMO-DB datasets. Each metric represents a different aspect of the model’s performance.

**Figure 9:**
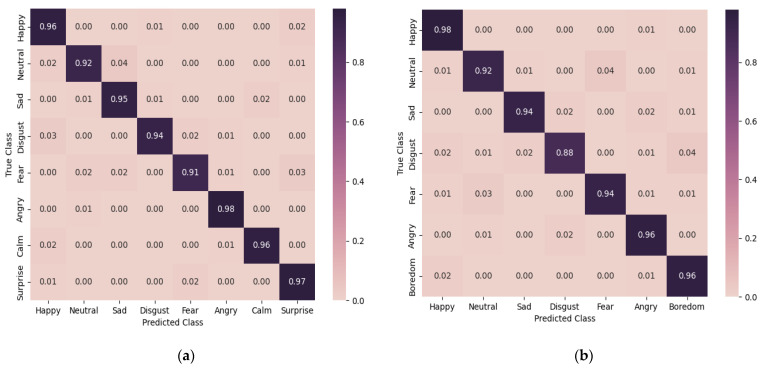
The confusion matrices on the (**a**) RAVDESS and (**b**) EMO-DB datasets.

The assessment was further carried out utilizing a confusion matrix, as depicted in Figure 9. This analytical tool provided visual representation and further elucidation of the model’s performance. It showcased that the model achieved an accuracy that transcended the threshold of 91% on the RAVDESS and 88% on the EMO-DB datasets for every distinct emotional category. This result points to a fairly elevated degree of precision in the classification tasks, denoting the model’s robust and reliable capacity for emotion categorization.

### 4.5. Discussion and Limitations

This methodological integration provided a more holistic approach toward understanding and interpreting speech emotions, demonstrating considerable promise and superior accuracy compared to existing models. However, a comprehensive discussion of the research necessitates acknowledgement of potential limitations and the scope of generalizability.

One limitation pertains to the incorporation of MFCCs and Speech2Vec for semantic feature extraction. MFCCs, while computationally efficient, primarily focus on acoustic characteristics, potentially missing some of the subtler emotional nuances found in prosody or speech intonation. On the other hand, Speech2Vec’s performance hinges on the quality and diversity of the training data. The model’s capacity to accurately capture the semantic properties of speech could be compromised in situations where the training data are not representative of the application context, or in scenarios involving multiple languages or distinct dialects. Thus, the robustness of the semantic feature extraction remains a subject for future investigation.

Regarding the LSTM-based deep learning architecture, while it excels in producing fixed-length embeddings and handling temporal dependencies, these advantages come at the cost of computational intensity. This might limit its applicability in real-time applications or in contexts with constrained computational resources. Additionally, like other deep learning models, the LSTM’s inherent “black box” nature presents an interpretability challenge. Understanding the model’s inner workings and decision-making processes can be complex, which might impede full comprehension of its performance and the potential for further refinement.

In terms of generalizability, it is crucial to exercise caution when extrapolating our results. The superior performance demonstrated by our model might be tied to the specific characteristics of our dataset, including the language, the quality and diversity of emotional states, and the number of speakers. To fully determine the robustness and universality of our model, it is imperative to test it on various independent datasets, across languages, and in different contextual scenarios.

Moreover, established benchmark datasets can exhibit various biases such as speech recognition [44], geographical and demographic [45], and temporal [46]. These biases can inadvertently influence the performance and generalizability of emotion classification or quantification models. These biases could limit the model’s ability to accurately and fairly recognize or quantify emotions “in the wild”, i.e., in diverse, real-world scenarios beyond the conditions represented in the training data. In our work, while we did not explicitly control for these biases in the dataset, we acknowledge their potential existence and the limitations they might impose on the generalizability of our results. Going forward, we aim to address these limitations by incorporating more diverse data in terms of geography, demographics, and time, thereby improving the robustness and applicability of our model in real-world, diverse settings.

Lastly, the incorporation of spectrograms for detecting emotional changes over time adds another layer of complexity to the model. While it contributes to a more comprehensive understanding of emotional fluctuations, the feature extraction process and interpretation of spectrograms can be challenging. Additionally, the temporal resolution of spectrograms may have an impact on the model’s performance, warranting further investigation.

Despite these limitations, our study marks a significant stride toward a more robust and semantically aware SER model. Future work should continue to address these issues, seeking more effective solutions for semantic feature extraction, improving model interpretability, and validating our findings across diverse real-world conditions. Through continual refinement, we aspire to develop a SER model that is not only academically innovative but also practically beneficial for a broad range of applications.

## 5. Conclusions and Future Scope

To create an effective SER model, it is necessary to tackle the difficult tasks of creating a suitable algorithm to obtain important speech characteristics that enhance the model performance and overcoming the challenges associated with obtaining and interpreting speech features to identify emotions, which are the primary obstacles to the development of an SER model. The difficulties in addressing these tasks have been alleviated to some extent by the progress made in contemporary deep-learning algorithms. Thus, this study demonstrated the efficacy of a novel approach to speech emotion recognition that leverages innovative techniques for feature extraction and a deep learning architecture for classification. The results indicate that the combination of fully CNN and MFCC with Speech2Vec features provides a more robust representation of speech data, leading to superior accuracy compared with existing baseline models. Furthermore, the use of an LSTM and a fully connected layer enabled the creation of a fixed-length embedding, simplifying the processing of speech signals and improving the accuracy of SER. These contributions are significant and have important implications for the future development of more effective speech recognition models. This study also provides several promising directions for future research. For instance, it proposes the integration of utterance-level features with the proposed system to improve its accuracy. Additionally, the integration of SER into a recommender system [47,48] is another promising avenue for further exploration because it can enhance the personalization and contextualization of recommendations. This integration can be achieved by leveraging SER to analyze the emotions and moods of users, which can then be used to tailor recommendations to their current emotional states. Furthermore, exploring the use of visual modalities in conjunction with audio data can also be an exciting area for future research, as visual cues, such as facial expressions and body language, can convey valuable emotional information. Overall, these research areas offer significant potential for advancing the field of SER and enhancing its practical applications.

## Figures and Tables

**Table 1:** Implementation specifications.

Model Implementation	RAM	128 GB
	GPU	GeForce RTX 3090 Ti, 24 GB GDDR6X, 384-bit
	CPU	AMD EPYC 7543 32-Core Processor
	Memory	SSD 1024 GB
	OS	Linux Ubuntu
	Programming environment	Python, Pytorch

## Data Availability

Not applicable.
